# Poly[(μ_3_-3,5-diisopropyl-4*H*-1,2,4-triazolato-κ^3^ *N*:*N*′:*N*′′)silver(I)]

**DOI:** 10.1107/S1600536814008083

**Published:** 2014-04-26

**Authors:** Guo-Gen Cui, Xiao-Xi Yang, Jian-Ping Yang, Xiang Jiang

**Affiliations:** a School of Chemistry and Chemical Engineering, South China University of Technology, Guangzhou 510640, People’s Republic of China

## Abstract

In the polymeric title compound, [Ag(C_8_H_14_N_3_)]_*n*_, the Ag^I^ cation is coordinated by three N atoms from three 3,5-diisopropyl-1,2,4-triazolate anions in a T-shaped geometry. The Ag^I^ cation deviates from the coordination plane by 0.014 (1) Å and the N—Ag—N bond angles are 96.85 (11), 97.72 (10) and 165.39 (12)°. The triazolate anion bridges three Ag^I^ cations, forming a three-dimensional polymeric network.

## Related literature   

For the synthesis, see: Yang *et al.* (2009 ▶). For related structures, see: Yang *et al.* (2007 ▶); Ling *et al.* (2012 ▶).
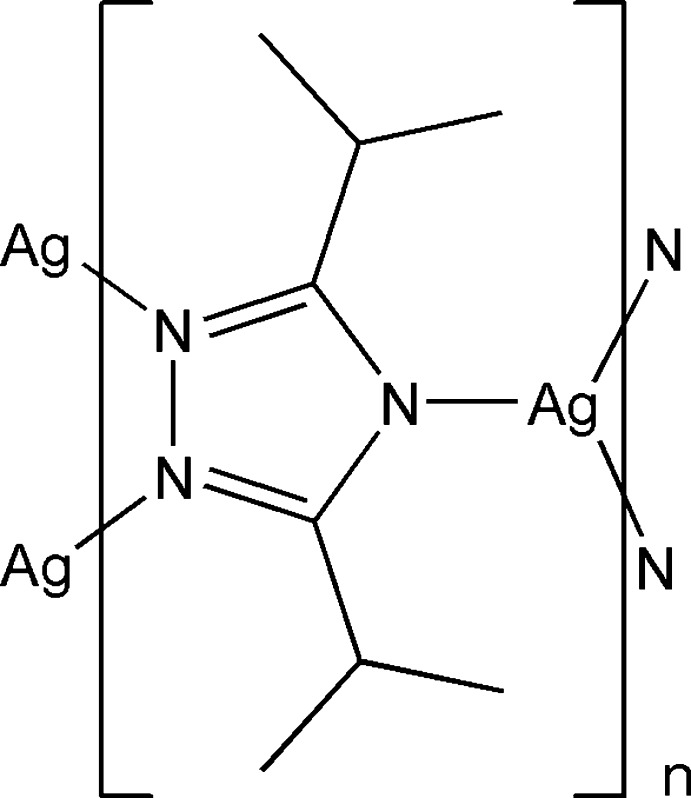


## Experimental   

### 

#### Crystal data  

[Ag(C_8_H_14_N_3_)]
*M* *_r_* = 260.09
Orthorhombic, 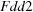

*a* = 20.853 (7) Å
*b* = 14.099 (5) Å
*c* = 14.364 (5) Å
*V* = 4223 (2) Å^3^
*Z* = 16
Mo *K*α radiation
μ = 1.86 mm^−1^
*T* = 296 K
0.20 × 0.15 × 0.10 mm

#### Data collection  

Bruker APEXII CCD diffractometer
Absorption correction: multi-scan (*SADABS*; Bruker, 2001 ▶) *T*_min_ = 0.707, *T*_max_ = 0.836
6151 measured reflections
1646 independent reflections
1626 reflections with *I* > 2σ(*I*)
*R*_int_ = 0.018

#### Refinement  

*R*[*F* ^2^ > 2σ(*F* ^2^)] = 0.017
*wR*(*F* ^2^) = 0.045
*S* = 1.08
1646 reflections
113 parameters
1 restraint
H-atom parameters constrained
Δρ_max_ = 0.35 e Å^−3^
Δρ_min_ = −0.25 e Å^−3^
Absolute structure: Flack (1983 ▶), 645 Friedel pairs
Absolute structure parameter: −0.02 (4)

### 

Data collection: *APEX2* (Bruker, 2007 ▶); cell refinement: *SAINT* (Bruker, 2007 ▶); data reduction: *SAINT*; program(s) used to solve structure: *SHELXTL* (Sheldrick, 2008 ▶); program(s) used to refine structure: *SHELXTL*; molecular graphics: *SHELXTL*; software used to prepare material for publication: *SHELXTL*.

## Supplementary Material

Crystal structure: contains datablock(s) I, global. DOI: 10.1107/S1600536814008083/xu5742sup1.cif

Structure factors: contains datablock(s) I. DOI: 10.1107/S1600536814008083/xu5742Isup2.hkl

CCDC reference: 996628

Additional supporting information:  crystallographic information; 3D view; checkCIF report

## Figures and Tables

**Table 1:** Selected bond lengths (Å)

Ag1—N1	2.135 (2)
Ag1—N2^i^	2.131 (3)
Ag1—N3^ii^	2.504 (3)

Symmetry codes: (i) 
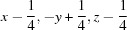
; (ii) 
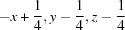
.

**Fig. 1.**
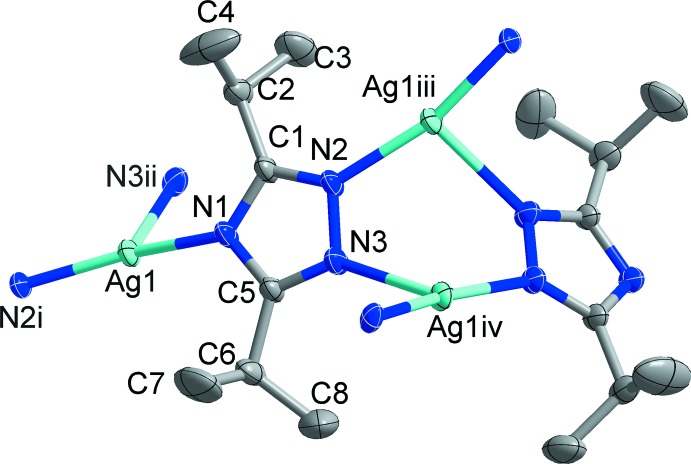
The coordination environment of the AgI cation in (I), showing the atom-labelling number. Displacement ellipsoids are drawn at the 30% probability level (Hydrogen atoms are omitted for clarity). [Symmetry codes: (i) 1.25 - x, -0.25 + y, -0.25 + z; (ii) -0.25 + x, 0.25 - y, -0.25 + z; (iii) 1.25 - x, 0.25 + y, 0.25 + z; (iv) 0.25 + x, 0.25 - y, 0.25 + z.]

**Fig. 2.**
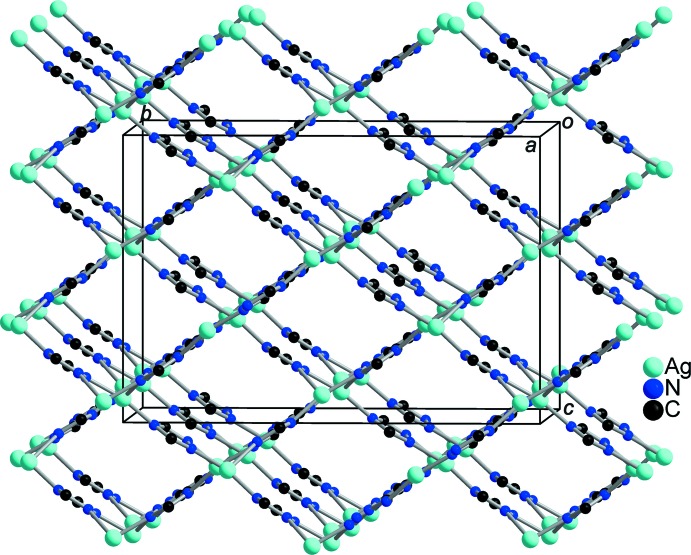
A view of the structure of (I), showing the three-dimensional network viewed along the a axis (the isopropyl groups are omitted for clarity).

## supplementary crystallographic information

### 1. Comment

The asymmetric unit of (I) contains one Ag(I) cation and one 3,5-diisopropyl-1,2,4-triazolate (diptrz) ligand. The coordination environment of the Ag (I) cation can be viewed as a "T" geometry, surrounded by three N atoms from three diptrz ligands (Fig. 1). The Ag1—N1 and Ag1—N2^i^ bond distances are 2.135 (3) and 2.131 (3) Å, respectively, agreement with those of Ag—N bond distances in reported 1,2,4-triazole-based silver compounds (Ling *et al.*, 2012). The Ag1—N3^ii^ bond distance is 2.504 (4) Å, agreeing well with those observed in 4-amino-3,5-diisopropyl-1,2,4-triazolate-based (4-NH_2_-3,5-iPr_2_-tz) silver compounds (Yang *et al.*, 2007). The diptrz ligand bridges neighboring three Ag(I) ions in a µ_3_-N^1^,*N*^2^,*N*^4^ fashion. The distance between Ag1^iii^ and Ag1^iv^ (bridged by diptrz in µ_1,2_-mode) is 3.3186 (11) Å, which is less than the sum of the van der Waals radii for the Ag atoms (3.44 Å). The infinite connection of Ag—N results the formation of three-dimensional compound (I) (Fig. 2).

### 2. Experimental

3,5-Diisopropyl-1*H*-1,2,4-triazole (Hdiptrz) was prepared according to the previous report (Yang *et al.*, 2009). Hdiptrz (0.046 g, 0.3 mmol) was dissolved in the mixture solution of acetonitrile (5 ml) and distilled water (5 ml). Then, silver acetate (0.075 g, 0.45 mmol) was added to the mixture. The mixture was stirred at room temperature for 10 min, and the white emulsion was obtained. The mixture was then transferred into a 15 ml Teflon-lined Parr bomb and heated at 413 K for 3 days. Then the reaction was cooled down to room temperature and block colourless crystals of (I) were obtained in 36% yield (based on Hdiptrz) by filtration.

### 3. Refinement

All H atoms were generated geometrically and allowed to ride on their parent atoms in riding-model approximations, with C—H = 0.96 Å and *U*_iso_(H) = 1.2*U*_eq_(C) for methyl H atoms, and C—H = 0.98 Å and *U*_iso_(H) = 1.2*U*_eq_(C) for methine H atoms.

### Crystal data

**Table:**

[Ag(C_8_H_14_N_3_)]	*D*_x_ = 1.636 Mg m^−^^3^
*M**_r_* = 260.09	Mo *K*α radiation, λ = 0.71073 Å
Orthorhombic, *F**d**d*2	Cell parameters from 6779 reflections
*a* = 20.853 (7) Å	θ = 2.9–30.8°
*b* = 14.099 (5) Å	µ = 1.86 mm^−^^1^
*c* = 14.364 (5) Å	*T* = 296 K
*V* = 4223 (2) Å^3^	Block, colorless
*Z* = 16	0.20 × 0.15 × 0.10 mm
*F*(000) = 2080	

### Data collection

**Table:**

Bruker APEXII CCD diffractometer	1646 independent reflections
Radiation source: fine-focus sealed tube	1626 reflections with *I* > 2σ(*I*)
Graphite monochromator	*R*_int_ = 0.018
φ and ω scans	θ_max_ = 25.2°, θ_min_ = 3.5°
Absorption correction: multi-scan (*SADABS*; Bruker, 2001)	*h* = −22→24
*T*_min_ = 0.707, *T*_max_ = 0.836	*k* = −16→16
6151 measured reflections	*l* = −15→17

### Refinement

**Table:**

Refinement on *F*^2^	Secondary atom site location: difference Fourier map
Least-squares matrix: full	Hydrogen site location: inferred from neighbouring sites
*R*[*F*^2^ > 2σ(*F*^2^)] = 0.017	H-atom parameters constrained
*wR*(*F*^2^) = 0.045	*w* = 1/[σ^2^(*F*_o_^2^) + (0.0255*P*)^2^ + 5.9277*P*] where *P* = (*F*_o_^2^ + 2*F*_c_^2^)/3
*S* = 1.08	(Δ/σ)_max_ < 0.001
1646 reflections	Δρ_max_ = 0.35 e Å^−^^3^
113 parameters	Δρ_min_ = −0.25 e Å^−^^3^
1 restraint	Absolute structure: Flack (1983), 645 Friedel pairs
Primary atom site location: structure-invariant direct methods	Absolute structure parameter: −0.02 (4)

### Special details

**Table:**

Experimental. Analysis calculated (found) for Ag(C_8_N_3_H_14_) (%): C, 36.94 (36.82); H, 5.43 (5.71); N, 16.16 (16.23)%. IR spectrum for (I) (KBr,, cm^-1^): 2962(*s*), 2925(*s*), 2871(*m*), 1498(*s*), 1466(*s*), 1435(*m*), 1380(*m*), 1361(*m*), 1301 (*m*), 1271(*m*), 1168(*m*), 1111(w), 1089(*m*), 1042(*m*), 774(w).
Geometry. All e.s.d.'s (except the e.s.d. in the dihedral angle between two l.s. planes) are estimated using the full covariance matrix. The cell e.s.d.'s are taken into account individually in the estimation of e.s.d.'s in distances, angles and torsion angles; correlations between e.s.d.'s in cell parameters are only used when they are defined by crystal symmetry. An approximate (isotropic) treatment of cell e.s.d.'s is used for estimating e.s.d.'s involving l.s. planes.
Refinement. Refinement of *F*^2^ against ALL reflections. The weighted *R*-factor *wR* and goodness of fit *S* are based on *F*^2^, conventional *R*-factors *R* are based on *F*, with *F* set to zero for negative *F*^2^. The threshold expression of *F*^2^ > σ(*F*^2^) is used only for calculating *R*-factors(gt) *etc*. and is not relevant to the choice of reflections for refinement. *R*-factors based on *F*^2^ are statistically about twice as large as those based on *F*, and *R*- factors based on ALL data will be even larger.

### Fractional atomic coordinates and isotropic or equivalent isotropic displacement parameters (Å^2^)

**Table:**

	*x*	*y*	*z*	*U*_iso_*/*U*_eq_	
Ag1	0.017808 (10)	0.114711 (15)	0.64610 (4)	0.04068 (9)	
N1	0.09085 (12)	0.1578 (2)	0.7413 (2)	0.0404 (6)	
N2	0.18000 (12)	0.1560 (2)	0.8229 (2)	0.0422 (6)	
N3	0.16637 (12)	0.25041 (19)	0.8030 (2)	0.0455 (7)	
C1	0.13441 (15)	0.1034 (2)	0.7855 (3)	0.0409 (8)	
C2	0.12967 (17)	−0.0027 (3)	0.7926 (3)	0.0569 (11)	
H2	0.0971	−0.0231	0.7477	0.068*	
C3	0.1906 (3)	−0.0505 (3)	0.7667 (5)	0.0918 (18)	
H3A	0.2247	−0.0270	0.8052	0.138*	
H3B	0.2002	−0.0376	0.7026	0.138*	
H3C	0.1862	−0.1176	0.7755	0.138*	
C4	0.1069 (4)	−0.0329 (4)	0.8876 (7)	0.141 (3)	
H4A	0.1046	−0.1009	0.8902	0.211*	
H4B	0.0652	−0.0066	0.8992	0.211*	
H4C	0.1364	−0.0106	0.9340	0.211*	
C5	0.11257 (13)	0.2480 (2)	0.7535 (2)	0.0394 (7)	
C6	0.07883 (18)	0.3347 (3)	0.7186 (3)	0.0560 (9)	
H6	0.0590	0.3188	0.6587	0.067*	
C7	0.0254 (3)	0.3612 (5)	0.7860 (7)	0.120 (3)	
H7A	0.0016	0.3053	0.8024	0.180*	
H7B	−0.0028	0.4063	0.7570	0.180*	
H7C	0.0436	0.3886	0.8412	0.180*	
C8	0.1231 (3)	0.4168 (4)	0.7028 (6)	0.120 (3)	
H8A	0.1429	0.4344	0.7606	0.180*	
H8B	0.0992	0.4696	0.6788	0.180*	
H8C	0.1557	0.3990	0.6588	0.180*	

### Atomic displacement parameters (Å^2^)

**Table:**

	*U*^11^	*U*^22^	*U*^33^	*U*^12^	*U*^13^	*U*^23^
Ag1	0.03118 (11)	0.04062 (12)	0.05024 (14)	−0.00108 (8)	−0.01624 (8)	−0.00135 (12)
N1	0.0305 (12)	0.0401 (14)	0.0505 (16)	0.0034 (11)	−0.0191 (12)	−0.0062 (13)
N2	0.0315 (12)	0.0409 (15)	0.0542 (17)	0.0038 (11)	−0.0152 (11)	−0.0026 (13)
N3	0.0370 (13)	0.0367 (14)	0.0629 (19)	0.0008 (11)	−0.0149 (13)	−0.0033 (13)
C1	0.0341 (16)	0.0395 (16)	0.049 (2)	0.0037 (13)	−0.0117 (14)	−0.0069 (15)
C2	0.0544 (19)	0.0364 (19)	0.080 (3)	0.0010 (18)	−0.024 (2)	−0.003 (2)
C3	0.092 (3)	0.054 (3)	0.129 (5)	0.021 (2)	−0.020 (3)	−0.029 (3)
C4	0.200 (7)	0.059 (3)	0.164 (7)	0.006 (4)	0.085 (7)	0.027 (5)
C5	0.0344 (16)	0.0379 (17)	0.0458 (19)	0.0037 (15)	−0.0113 (12)	−0.0059 (16)
C6	0.051 (2)	0.047 (2)	0.070 (3)	0.0063 (17)	−0.0288 (18)	0.0000 (19)
C7	0.104 (5)	0.092 (4)	0.164 (8)	0.060 (4)	0.018 (5)	0.017 (5)
C8	0.096 (4)	0.077 (4)	0.188 (8)	−0.004 (3)	−0.019 (4)	0.064 (5)

### Geometric parameters (Å, º)

**Table:**

Ag1—N1	2.135 (2)	C3—H3B	0.9600
Ag1—N2^i^	2.131 (3)	C3—H3C	0.9600
Ag1—N3^ii^	2.504 (3)	C4—H4A	0.9600
Ag1—Ag1^iii^	3.3187 (11)	C4—H4B	0.9600
N1—C1	1.349 (4)	C4—H4C	0.9600
N1—C5	1.361 (4)	C5—C6	1.497 (5)
N2—C1	1.320 (4)	C6—C8	1.498 (7)
N2—N3	1.391 (4)	C6—C7	1.523 (9)
N2—Ag1^iv^	2.131 (3)	C6—H6	0.9800
N3—C5	1.329 (4)	C7—H7A	0.9600
N3—Ag1^v^	2.504 (3)	C7—H7B	0.9600
C1—C2	1.502 (5)	C7—H7C	0.9600
C2—C3	1.485 (6)	C8—H8A	0.9600
C2—C4	1.507 (9)	C8—H8B	0.9600
C2—H2	0.9800	C8—H8C	0.9600
C3—H3A	0.9600		
			
N2^i^—Ag1—N1	165.39 (12)	H3B—C3—H3C	109.5
N2^i^—Ag1—N3^ii^	96.85 (11)	C2—C4—H4A	109.5
N1—Ag1—N3^ii^	97.72 (10)	C2—C4—H4B	109.5
N2^i^—Ag1—Ag1^iii^	70.99 (8)	H4A—C4—H4B	109.5
N1—Ag1—Ag1^iii^	115.93 (8)	C2—C4—H4C	109.5
N3^ii^—Ag1—Ag1^iii^	59.70 (6)	H4A—C4—H4C	109.5
C1—N1—C5	104.3 (2)	H4B—C4—H4C	109.5
C1—N1—Ag1	128.3 (2)	N3—C5—N1	111.9 (3)
C5—N1—Ag1	125.9 (2)	N3—C5—C6	123.8 (3)
C1—N2—N3	107.9 (2)	N1—C5—C6	124.3 (3)
C1—N2—Ag1^iv^	138.0 (2)	C5—C6—C8	113.0 (4)
N3—N2—Ag1^iv^	114.09 (19)	C5—C6—C7	109.3 (4)
C5—N3—N2	105.0 (3)	C8—C6—C7	111.0 (5)
C5—N3—Ag1^v^	139.9 (2)	C5—C6—H6	107.8
N2—N3—Ag1^v^	113.20 (19)	C8—C6—H6	107.8
N2—C1—N1	110.9 (3)	C7—C6—H6	107.8
N2—C1—C2	125.4 (3)	C6—C7—H7A	109.5
N1—C1—C2	123.7 (3)	C6—C7—H7B	109.5
C3—C2—C1	112.2 (3)	H7A—C7—H7B	109.5
C3—C2—C4	111.6 (5)	C6—C7—H7C	109.5
C1—C2—C4	111.3 (4)	H7A—C7—H7C	109.5
C3—C2—H2	107.1	H7B—C7—H7C	109.5
C1—C2—H2	107.1	C6—C8—H8A	109.5
C4—C2—H2	107.1	C6—C8—H8B	109.5
C2—C3—H3A	109.5	H8A—C8—H8B	109.5
C2—C3—H3B	109.5	C6—C8—H8C	109.5
H3A—C3—H3B	109.5	H8A—C8—H8C	109.5
C2—C3—H3C	109.5	H8B—C8—H8C	109.5
H3A—C3—H3C	109.5		

Symmetry codes: (i) *x*−1/4, −*y*+1/4, *z*−1/4; (ii) −*x*+1/4, *y*−1/4, *z*−1/4; (iii) −*x*, −*y*, *z*; (iv) *x*+1/4, −*y*+1/4, *z*+1/4; (v) −*x*+1/4, *y*+1/4, *z*+1/4.
